# Cancer symptom control trials: how may we advance this field?

**DOI:** 10.3747/co.2007.116

**Published:** 2007-06

**Authors:** N. MacDonald

**Canadian Institutes of Health: Research definition of palliative care:** Palliative care aims to improve the life of patients and families through the early identification and impeccable management of suffering associated with advanced illness and emphasis on the positive aspects of life inclusive of physical, psychosocial and spiritual sources. Palliative care is an exercise in prevention—prevention of suffering through prioritizing the diagnosis and skillful care of sources of distress throughout the course of illness and for the family into the bereavement period. It is not simply an end of life concept separate from other aspects of research and control. Palliative care research focuses on fundamental symptom mechanisms as well as the experience of the patient and the family.

—Adapted, with thanks, from the World Health Organization

The preceding palliative care definition, endorsed by the Canadian Institutes of Health Research (CIHR) Institute of Cancer Research, emphasizes early identification of sources of suffering in cancer patients and, by inference, the importance of clinical trials on symptom-control therapies introduced early in a patient’s course of illness. This noble objective is fully in keeping with the reports of an influential panel which recommends that patients and families have access to the full range of care measures at disease onset 1.

Today, few would disagree with the concept that therapy for symptom and psychosocial problems should be integrated with traditional measures of curing or controlling cancer (surgery, radiotherapy, chemotherapy). However, model programs blending these elements of care from diagnosis are still not common.

Cancer centres may be making progress in moving from rhetoric to reality, but slowly in the clinical research sphere. For example, anorexia–cachexia is often encountered early in the course of the illness, but research on the problem is modest. In 2005, 15 of 4917 abstracts published by the American Society of Clinical Oncology were concerned with cancer nutrition, including cancer cachexia. Only a few of these were clinical trials, with small patient enrolment. No progress was seen in 2006, with its 10 nutrition presentations. Research presentations on the problems of chronic symptoms—aside from those on pain (a dramatic, acute source of suffering demanding immediate attention)—continue to lag.

Because of the common presence of cancer anorexia–cachexia as a source of patient–family distress, and because of its devastating effects (loss of function, and consequent dependency and poorer prognosis), I will use that symptom as a theme to illustrate problems in conducting clinical studies that need to enlist patients early in the course of their illness.

## DIFFICULTIES WITH TRIAL ENROLMENT AND DROPOUT

I recently served as the Canadian Principal Investigator (pi) for an international anorexia trial. Canadian patients represented approximately 25% of the patients enrolled in that study 2, but most came from my home institution, one other academic centre, and a community cancer centre; enrolment from our largest cancer centres was poor. When the local investigators were asked about their low enrolment, they replied that they had had difficulty in keeping the trial “front and centre” with their oncology colleagues. Failure to recommend patients for the trial until cachexia was far advanced was also a problem. Another major reason expressed for the delay in trial consideration was competition with conventional cancer chemoradiotherapy trials.

Why “competition”?

Most clinical trials are sponsored by pharmaceutical firms. For example, in 2005, our Institutional Review Board at McGill University received 42 oncology studies, of which 30 were industry-sponsored submissions. Sponsoring companies are usually interested in conducting trials with narrow inclusion criteria. These “fastidious” trials (defined as “using homogeneous groups, reducing or eliminating ambiguity” 3) are designed to examine the use of a specific therapy in a carefully controlled environment, leaving little doubt that any observed efficacy directly relates to the tested product. This aim is sensible, and expected by regulatory authorities, but it leads to a trial milieu that may not reflect the general practice of oncology. “Pragmatic” trials—defined as those that “would incorporate heterogeneity, occasional or frequent ambiguity, and other ‘messy’ aspects of clinical practice” 3 are more relevant to actual oncology practice, but are less commonly conducted. The protocols for fastidious trials usually contain an exclusion clause with wording along the following lines: “Concomitant employment of other experimental therapies during the course of the clinical trial is prohibited” 3.

Inherently, a paradox is evident. The cancer community believes that research into palliation and symptom control deserves a high priority 4 and that that research should commence with the onset of a symptom (for example, as soon as a weight-losing patient with lung cancer presents with disease). However, the same patient cannot go on a symptom control trial if enrolled in a chemotherapy study, because participation in another clinical trial is proscribed. Consequently, late in the course of illness, a small coterie of exhausted patients with profound weight loss may now be asked if they wish to consider enlisting in a cachexia trial. But is this trial hierarchy logical?

Exclusion of patients from participation in two simultaneous trials is usually justified for these reasons:

- Regulatory authorities will not permit simultaneous trials.
- Participation in two trials will present confounding information that will make it impossible for the pharmaceutical firm to properly study the efficacy of the drug or drug and radiotherapy combination of interest and to accurately assign adverse reactions.

These excellent reasons fail to take note of certain confounding facts. Many patients with advanced cancer are using a variety of untested combinations of diet, supplements, vitamins, herbs, naturopathic products, yoga, and so on 5. Pharmaceutical trials have not usually controlled for these widespread potential confounders. Moreover, the populations at risk for cancer tend to be older, and they often have comorbid conditions—notably diabetes, hypertension, cardiovascular disease, and chronic obstructive pulmonary disease. Patients with these disorders are thus receiving a wide variety of drugs that could affect disease activity 6–8 and certainly may interfere with drug metabolism. Information on these agents and their relevance usually does not appear in study reports.

Excluding careful clinical research observations on agents aimed at helping control symptoms, while ignoring the chaotic, uncontrolled potential effects of alternative and non-cancer-related therapies on drug and radiotherapy response is therefore illogical. From a statistical point of view, patients who are also engaged in a symptom control trial can be readily stratified, although trials may then need to be larger.

We worship at the “evidence based” altar. Many of our symptom control trials are lightly regarded because of small patient enrolment and high dropout rates from death and increasing frailty. Here are some examples from two recent studies published by leaders in the anorexia–cachexia field:

- Fearon and colleagues 9 recently reported on the efficacy of two doses of an omega-3 fatty acid on pancreatic cancer–induced cachexia. A robust effect was not found, only a favourable trend at the level of a 2-g dose of eicosapentaenoic acid. The mean weight loss on trial entrance was 18%, and the dropout rate among these profoundly ill patients was approximately 50% over 8 weeks.
- The recent article by Strasser *et al.* 10 on cannabinoid use in combating anorexia had a 32% dropout rate in 8 weeks; the study population had a mean weight loss of 11.9% upon enrolment.

We will not advance cachexia research until we can invite people with early weight loss to consider trial participation.

## OBLIGATION TO DEVELOPING COUNTRIES

Recent advances have come at a cost—a cost passed on to the health system, or now, frequently, to the patient as governments lag in picking up the tab for non-curative, extraordinarily expensive therapies that may modestly prolong survival for a select group of patients. To paraphrase the Etruscan general Pyrrhus’ comment on winning a battle with the Romans at great cost to his army, “A few more pharmaceutical advances like cetuximab and our cancer care system is bankrupt.”

The costs of new anticancer agents create major dilemmas in Canada. What about resource-poor countries such as those of the African continent? Those countries will never be able to pay for drugs like bevacizumab or cetuximab. Do we have an ethical obligation to carry out clinical trials using inexpensive agents which, while not commercially profitable, may be more available to poor countries?

Recent ideas for cachexia trials include, among others, combinations of inexpensive anti-inflammatory agents with stimulants of muscle synthesis. If this research is successful, the fruits could be widely used. Moreover, the outcomes of these “pragmatic trials” may more readily predict response in a general cancer population. Our research sponsors, including the pharmaceutical industry, must consider the plight of cancer patients in poor lands and direct some research support into studies on agents that can be more readily accessed.

## CONCLUSION

On ethical and scientific grounds alike, exclusion of options for participation in symptom control trials by newly diagnosed patients does not stand up to scrutiny. I suggest that the following clinical trial policies are worthy of consideration:

- In the absence of clear ethical or scientific reasoning, patients enrolled in chemotherapy and radiotherapy trials should be able, at the same time, to participate in symptom control trials. If the sponsoring company or pi demands that the exclusion clause barring patients from symptom control studies must stand, the onus should be on the company or the pi to provide evidence that this demand is ethically and scientifically justified.
- If symptom control studies are available to patients presenting at any stage of their disease, it is imperative that an investigator fully inform the patients of the opportunity for participation in symptom control trials in addition to other trials under discussion.
- A new trial hierarchy should be established wherein trials with probable major impact on tumour response rank at the top (as they now do).
  - The second rank should include pragmatic trials in which patients may receive chemoradiotherapeutic study agents and still enrol in symptom studies—that is, pragmatic trials should receive a higher ranking than they do at present.
  - The third rank could include fastidious trials.
  - In the fourth rank are the “me too” trials—the ones that are often lucrative, but that are not providing much in the way of new knowledge. A variant of an existing agent usually has similar properties and probable modest efficacy and safety distinctions. These trials should not, however, congest the clinical trials structure.
